# High Incidence of Pulmonary Tuberculosis a Decade after Immigration, Netherlands

**DOI:** 10.3201/eid1004.030530

**Published:** 2004-04

**Authors:** Annelies M. Vos, Abraham Meima, Suzanne Verver, Caspar W.N. Looman, Vivian Bos, Martien W. Borgdorff, J. Dik F. Habbema

**Affiliations:** * Erasmus MC, University Medical Center Rotterdam, Rotterdam, the Netherlands; † KNCV Tuberculosis Foundation, The Hague, the Netherlands

**Keywords:** Tuberculosis, pulmonary, incidence, migrants, screening, Netherlands

## Abstract

Incidence rates of pulmonary tuberculosis among immigrants from high incidence countries remain high for at least a decade after immigration into the Netherlands. Possible explanations are reactivation of old infections and infection transmitted after immigration. Control policies should be determined on the basis of the as-yet unknown main causes of the persistent high incidence.

We describe patterns of incidence rates of pulmonary tuberculosis in immigrants in the Netherlands according to the length of time since immigration. Insight in these patterns is needed to evaluate tuberculosis control policies that aim to reduce transmission. The Dutch control policy differs from policies in other industrialized countries: not only is obligatory screening by chest x-ray performed at the time of immigration, but immigrants are also invited for voluntary follow-up screening at 6-month intervals in the first 2 years after immigration.

## The Study

We performed a retrospective cohort analysis of all legal immigrants notified as having pulmonary tuberculosis in the Netherlands between 1996 and 2000; pulmonary tuberculosis referred to any form of active tuberculosis that involved the lungs. Patient data were obtained from the Netherlands Tuberculosis Register and included date of birth, date of arrival in the Netherlands, time of diagnosis, localization of tuberculosis, country of origin, and sex. To account for fact that the reported time of immigration was often exactly 1, 2, 3, . . . years before diagnosis (“digit preference”), time since immigration was categorized with boundaries well apart from the preferred digits (Table).

**Table:** Incidence rate and relative risk of pulmonary tuberculosis according to time since immigration, country of origin, age, sex, and year of diagnosis for immigrants in the Netherlands, 1996–2000

	*Incidence rate/100,000* *person-years (cases)*	*Multivariate relative* *risk (95% CI)*
Time since immigration (y) 0.5–1.4 1.5–2.4 2.5–3.4 3.5–4.4 4.5–6.4 6.5–9.4 9.5–19.4 >19.5	59 (292) 44 (169) 55 )166) 43 (118) 42 (245) 34 (247) 21 (338) 15 (430)	1.39 (1.14 to 1.69) 1.00 1.14 (0.91 to 1.43) 0.88 (0.69 to 1.11) 0.89 (0.72 to 1.09) 0.80 (0.65 to 0.98) 0.58 (0.48 to 0.71) 0.49 (0.40 to 0.60)
Country of origin Morocco Somalia Other Africa Turkey Asia Suriname and Antilles Latin America Central and Eastern Europe Other countries	47 (334) 373 (392) 69 (270) 21 (178) 25 (419) 16 (194) 19 (33) 22 (100) 5 (86)	1.83 (1.57 to 2.14) 11.30 (9.63 to 13.25) 2.14 (1.82 to 2.52) 0.83 (0.69 to 1.00) 1.00 0.68 (0.57 to 0.81) 0.76 (0.53 to 1.09) 0.74 (0.59 to 0.93) 0.21 (0.16 to 0.26)
Age (y) 0–14 15–24 25–34 35–44 45–54 55–64 >65 Sex Male Female Y of diagnosis 1996 1997 1998 1999 2000	13 (78) 45 (412) 39 (661) 28 (424) 17 (185) 17 (117) 19 (128) 37 (1,291) 20 (714) 31 (413) 30 (408) 25 (356) 28 (408) 27 (421)	0.25 (0.20 to 0.32) 1.00 (0.88 to 1.13) l.00 0.99 (0.87 to 1.12) 0.81 (0.68 to 0.97) 0.87 (0.71 to 1.08) 1.32 (1.05 to 1.64) 1.62 (1.48 to 1.78) l.00 1.00 0.97 (0.85 to 1.12) 0.80 (0.70 to 0.93) 0.87 (0.76 to 1.00) 0.83 (0.73 to 0.96)
		

Data on the number of immigrants residing in the Netherlands were obtained from the Organization for Reception of Asylum Seekers (COA) and from municipal population registers (GBA) as provided by Statistics Netherlands. Person-years at risk for pulmonary tuberculosis were first calculated separately for both the COA and GBA registers. Privacy regulations prohibit matching of the two datasets. Since asylum seekers are allowed to register themselves in the GBA after 1 year of stay in the Netherlands, overlap between the two registers had to be accounted for. We assumed that the percentage of asylum seekers registered twice increased linearly from an initial 0% of asylum seekers in the COA register during the first 6 months after immigration, to 80% at 3.5 years after immigration. We recognize the arbitrariness of this assumption. Therefore, we carried out a sensitivity analysis with contrasting assumptions—asylum seekers were never versus always registered twice—to assess the consequences of the uncertainty regarding double registrations. This did not alter the conclusions (results not shown).

By the end of 2000, close to two million immigrants were residing in the Netherlands, of a total population of nearly 16 million. Among the immigrant population, 2,661 patients with pulmonary tuberculosis patients were identified during 1996–2000. Information about country of origin and time since immigration was missing in 3% and 13% of the study patients, respectively, and was accounted for by multiple imputation (five times) to avoid bias in the calculation of incidence rates, relative risks, and confidence intervals (*1*). For country of origin and time since immigration, all information presented is based on the average number of cases in the imputed datasets.

Incidence rates were only calculated for the 2,005 patients in whom tuberculosis was diagnosed more than half a year after immigration because many patients with a case diagnosed within 6 months may already have had active tuberculosis at the time of immigration. These patients should be considered prevalent rather than incident cases.

The Figure shows that incidence rates decreased after 0.5–1.4 years since immigration for immigrants from most of the countries. Subsequently, the incidence rates were mostly stable from 1.5 to 9.4 years since immigration for the countries with initial incidence rates above or around 50/100,000 (as a general rule, immigrants from countries with incidence rates above this level are eligible for screening). African immigrants, especially Somalis, had the highest incidence rates. Since few Somalis immigrated before 1991, the observed increase in incidence rates >9.4 years after immigration has wide confidence intervals. In contrast to the incidence rates for most of the countries, incidence rates for immigrants from Surinam and the Netherlands Antilles were initially low and significantly increased after an initial decrease. Average incidence rates after immigration varied from 379/100,000 in Somalis to 5/100,000 in immigrants from the category “other countries” (Table). For comparison, the current incidence rate of pulmonary tuberculosis in the indigenous Dutch population is approximately 3/100,000.

**Figure:**
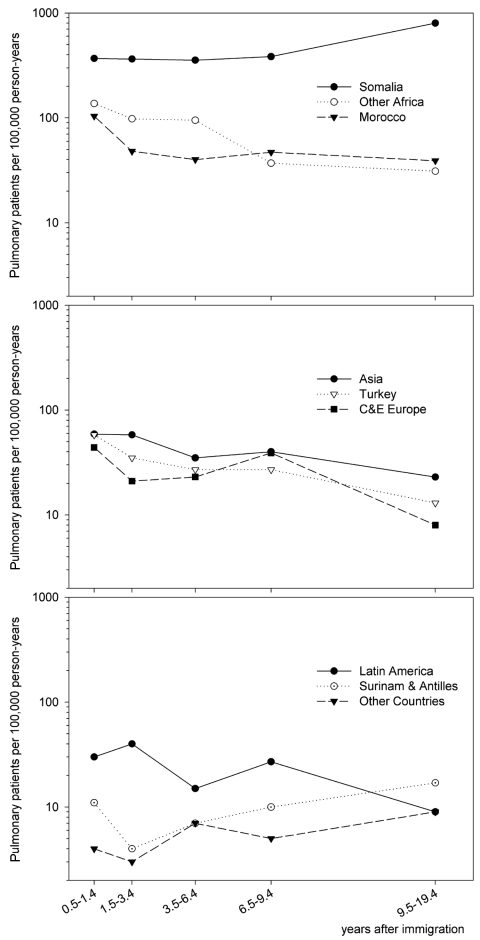
Incidence rates after immigration, according to country of origin. Central and Eastern (C&E) Europe includes Cyprus and the former Soviet Union.

Univariate and multivariate Poisson regression were performed by using Stata (Stata Corp, College Station, TX). For each imputed dataset, all risk factors were significant in the multivariate regression. The Table provides the combined multivariate results. A clear pattern in incidence rates was not observed in the first 3.4 years after immigration, but overall the incidence rates gradually decreased as time since immigration increased. Nonetheless, compared to 1.5–2.4 years, the incidence rate for 9.5–19.4 years since immigration had decreased by only 42%. Fifty-eight percent of patients, including those in whom tuberculosis was detected in the first 6 months, were found more than 2.5 years after immigration to the Netherlands, and 29% were found after more than 9.5 years.

As often observed, we found considerably lower incidence rates for children than for young adults and a significantly higher rate for males than females. Except for age, the univariate incidence rate ratios were largely similar to the multivariate ratios. In univariate analysis, incidence rate ratios in adults decreased with age, whereas in multivariate analysis the oldest age group had an increased risk. This result is due to confounding with country of origin and time since immigration: African immigrants had the highest incidence rates, but relatively few of them were older than 65 years, and they had immigrated relatively recently. Statistically significant, but small, differences in incidence rates according to year of diagnosis were observed (Table).

## Discussion

Our study shows that, in spite of a gradual decrease, the incidence rates of pulmonary tuberculosis in immigrants remain high even a decade after immigration. The persistent high incidence rates are consistent with results of previous studies (*2**–**5*). Our study combines data on all immigrant patients in whom tuberculosis was detected and all legal immigrants present in a 5-year period in a low incidence country, enabling detailed analysis with a long follow-up period.

We did not find a steep decline in incidence rates after immigration. One might anticipate such a decline, since the proportion of recently infected or reinfected persons will be higher sooner after immigration than later due to relatively low levels of transmission in the Netherlands. Recent infection is a known risk factor for developing active tuberculosis (*6**,**7*). Several explanations may account for the absence of an initial steep decline in incidence rates. First, the proportion of immigrants who were recently infected or reinfected may already have been low at the time of immigration. Next, the risk of reactivation of latent tuberculosis infection in these immigrants may have been higher than previously modeled in white nonimmigrant populations (*8**,**9*). Finally, immigrants residing in the Netherlands may have acquired new infections or reinfections, either through transmission within the Netherlands or through frequent visits to their country of origin. DNA fingerprinting data suggest that transmission within the Netherlands may indeed have occurred, although it is not the key factor; in a recent study, infections in 30% to 40% of Turkish, Moroccan, and Somali patients could be attributed to recent transmission, but 58% of all immigrant patients were not part of a cluster (*10*).

The Dutch screening policy consists of mandatory screening of immigrants at entry and voluntary screening in the next 2 years. Less than 50% of immigrants undergo voluntary screening in the second year (*11*). Screening identified 41% of the patients with a case diagnosed from 0.5 to 2.4 years after immigration. Screening may have influenced the observed incidence pattern slightly by diagnosing cases earlier than in the absence of screening. However, the average delay in detecting tuberculosis in immigrant patients who seek medical care themselves (passive detection) in the Netherlands is <3 months (*12*), and several studies reported upon by Toman (*13*) suggest that the period in which tuberculosis is detectable by x-ray, but has not yet led to clinical symptoms (preclinical detectable phase), is <6 months. Thus the incidence pattern in the first view years after immigration would not be very different in the absence of screening. The possible influence of screening on transmission has apparently not resulted in a pronounced downward trend in incidence rates over time: they would only have remained somewhat higher without screening.

In many industrialized countries, an increasing proportion of tuberculosis patients are immigrants. Immigrants account for >50% of the incidence in the Netherlands (*12*). Control policies with regard to immigrant tuberculosis usually rely on chest x-ray screening and treatment of active tuberculosis. A supplemental approach, recommended by the Institute of Medicine (*14*), is to conduct tuberculin skin testing and to apply preventive treatment of latent infections. Whether all tuberculin skin test–positive immigrants should be treated, or only selected high-risk groups such as immigrants with radiographic evidence of inactive disease, is under debate (*15*). Adhering to preventive treatment is also a point to consider (*15*). To answer the question of why the incidence rates remain high, the relative importance of three factors needs to be established: reactivation of old infections, transmission in the host country, and infections acquired during visits to the countries of origin. These answers are essential to evaluate the cost-effectiveness of the Dutch screening policy and of alternative options, including other screening policies and use of preventive treatment.
